# Optimized Informed Consent for Psychotherapy: Protocol for a Randomized Controlled Trial

**DOI:** 10.2196/39843

**Published:** 2022-09-30

**Authors:** Leonie Gerke, Sönke Ladwig, Franz Pauls, Manuel Trachsel, Martin Härter, Yvonne Nestoriuc

**Affiliations:** 1 Clinical Psychology Helmut-Schmidt-University/University of the Federal Armed Forces Hamburg Hamburg Germany; 2 Institute of Biomedical Ethics and History of Medicine University of Zurich Zurich Switzerland; 3 Clinical Ethics Unit University Hospital Basel Basel Switzerland; 4 Clinical Ethics Unit University Psychiatric Clinics Basel Basel Switzerland; 5 Department of Medical Psychology and Institute of Psychotherapy Center for Psychosocial Medicine University Medical Center Hamburg-Eppendorf Hamburg Germany; 6 Department of Systems Neuroscience University Medical Center Hamburg-Eppendorf Hamburg Germany

**Keywords:** expectation management, psychiatry, risks and side effects of psychotherapy, risk, counseling, consent, shared decision-making, decision-making, ethics, nocebo effect, side effect, adverse effect, psychotherapy, mental health, nocebo

## Abstract

**Background:**

Informed consent is a legal and ethical prerequisite for psychotherapy. However, in clinical practice, consistent strategies to obtain informed consent are scarce. Inconsistencies exist regarding the overall validity of informed consent for psychotherapy as well as the disclosure of potential mechanisms and negative effects, the latter posing a moral dilemma between patient autonomy and nonmaleficence.

**Objective:**

This protocol describes a randomized controlled web-based trial aiming to investigate the efficacy of a one-session optimized informed consent consultation.

**Methods:**

The optimized informed consent consultation was developed to provide information on the setting, efficacy, mechanisms, and negative effects via expectation management and shared decision-making techniques. A total of 122 participants with an indication for psychotherapy will be recruited. Participants will take part in a baseline assessment, including a structured clinical interview for Diagnostic and Statistical Manual of Mental Disorders-fifth edition (DSM-5) disorders. Eligible participants will be randomly assigned either to a control group receiving an information brochure about psychotherapy as treatment as usual (n=61) or to an intervention group receiving treatment as usual and the optimized informed consent consultation (n=61). Potential treatment effects will be measured after the treatment via interview and patient self-report and at 2 weeks and 3 months follow-up via web-based questionnaires. Treatment expectation is the primary outcome. Secondary outcomes include the capacity to consent, decisional conflict, autonomous treatment motivation, adherence intention, and side-effect expectations.

**Results:**

This trial received a positive ethics vote by the local ethics committee of the Center for Psychosocial Medicine, University-Medical Center Hamburg-Eppendorf, Hamburg, Germany on April 1, 2021, and was prospectively registered on June 17, 2021. The first participant was enrolled in the study on August 5, 2021. We expect to complete data collection in December 2022. After data analysis within the first quarter of 2023, the results will be submitted for publication in peer-reviewed journals in summer 2023.

**Conclusions:**

If effective, the optimized informed consent consultation might not only constitute an innovative clinical tool to meet the ethical and legal obligations of informed consent but also strengthen the contributing factors of psychotherapy outcome, while minimizing nocebo effects and fostering shared decision-making.

**Trial Registration:**

PsychArchives; http://dx.doi.org/10.23668/psycharchives.4929

**International Registered Report Identifier (IRRID):**

DERR1-10.2196/39843

## Introduction

Obtaining patients’ informed consent represents a legal and ethical obligation for conducting psychotherapy, which is embedded in numerous codes of conduct of international psychological institutions (eg, American Psychological Association [1], European Federation of Psychologists’ Associations [2]). Psychotherapists are legally bound to disclose information about the treatment, including all circumstances that might be essential for an autonomous decision (eg, § 630e, German Civil Code [3]). In an ethical framework, psychotherapists should strive to balance the 4 moral principles of respect for autonomy, beneficence, nonmaleficence, and justice [4]. The major components of a truthful informed consent are (1) the decision-making capacity of the patient, (2) disclosure of treatment information, (3) voluntariness, (4) patient understanding, and (5) the explicit statement of consent [4,5].

A key challenge in obtaining truthful informed consent is the required disclosure of the potential negative effects of psychotherapy. Balancing the principles of autonomy and nonmaleficence causes an ethical dilemma [6]. On the one hand, psychotherapists strive to provide transparent information about possible treatment risks to enable autonomous informed decision-making. On the other hand, the disclosure of risk information can be harmful in itself, as it might cause nocebo effects. The latter effects are usually described as adverse effects that are not caused by the procedure but by negative expectations or negative prior learning experiences [6,7]. Thus, patients who were initially informed about the potential side effects at the beginning of psychotherapy are suggested to be more likely to experience these disclosed side effects than patients who did not receive this information before.

In clinical practice, informed consent procedures often fall short of legal and ethical recommendations of truthful informed consent [8,9]. So far, informed consent does not seem to be an integral part of clinical routine [10,11]. Empirical research, however, provides evidence that patients experiencing mental disorders have extensive information and decision-making needs [12,13]. Thus, a one-size-fits-all approach for providing consent information that neglects individual information needs may be insufficient.

Recent research suggests that the informed consent procedure might be underestimated in its clinical relevance to strengthen the contributing factors of psychotherapy outcome [5]. There are already first indications that the disclosure of transparent and contextualized information can effectively optimize treatment expectations [14]. Since treatment expectations are considered a key mechanism of change in psychotherapy [15], it is conceivable that truthful informed consent might elicit relevant expectation effects that reinforce treatment outcome [5,16]. Moreover, the autonomous treatment motivation and treatment adherence of patients might be strengthened [17,18]. However, it remains unclear how these key predictors of psychotherapy outcome can be optimally addressed in a tangible informed consent procedure.

Framing, contextualization, and shared decision-making might represent 3 promising strategies to optimize informed consent procedures. First, positive information framing might contribute to overcoming the dilemma of presenting transparent information about risks of psychotherapy on the one hand and preventing nocebo effects on the other hand [19]. Empirical findings suggest that positive framing may reduce side-effect expectations [14] and nocebo side effects [20]. Second, contextualizing information might positively influence treatment expectations and reduce decisional conflicts [6,14]. Third, integrating shared decision-making strategies might promote patient-centered care [21,22]. Advanced approaches for addressing, integrating, and implementing those strategies within an elaborate informed consent procedure are still missing in clinical practice to this day [20,23].

In summary, 3 major research gaps can be identified. First, empirical data about whether and how psychotherapists obtain informed consent in clinical practice are sparse. In particular, the integration of risk information has not been investigated so far. Second, there is a lack of concrete implementation strategies for an informed consent procedure that simultaneously accounts for legal, ethical, and clinical functionalities. Third, the effects of informed consent procedures on factors contributing to psychotherapy outcomes have not yet been investigated systematically. The latter 2 research gaps will be specifically targeted in this study. An optimized informed consent consultation (OIC) for psychotherapy has been developed as a new clinical tool based on the most recent empirical evidence. OIC will be applied in a web-based context for 2 reasons: (1) to increase accessibility and (2) to reduce health risks due to the ongoing pandemic. As the web-based context requires access to the internet for all participants and the study staff, potential interferences due to internet connection problems will be considered by providing clear instructions to participants with backup weblinks and contact information via phone.

This study aims to investigate the efficacy of OIC for persons with an indication for psychotherapy. We hypothesize that treatment expectations, autonomous treatment motivation, and adherence intention increase to a greater extent in the OIC condition than in the treatment as usual (TAU) condition from baseline (T0) to the follow-up assessment (T2). Moreover, we assume that decisional conflicts and expectations about the side effects of psychotherapy decrease to a greater extent in the OIC condition than in the TAU condition from baseline (T0) to the follow-up assessment (T2).

## Methods

### Study Design

A randomized controlled superiority trial will be conducted in the web-based context. Participants with an indication for psychotherapy are equally assigned to one of the 2 trial conditions (either to OIC + TAU or to TAU alone). The trial includes 2 web-based study visits with an interval of 2 weeks between the visits and 2 follow-up assessments 2 weeks and 3 months later. Web-based study visits are conducted via RED connect, an internet platform providing video consultations compliant with the German Data Protection Directive. Written study information, informed consent, and questionnaire-based assessments are provided via the web-based software EFS Survey, which fulfills the international guidelines for information security (ISO 27001). This study has been designed in line with the Consolidated Standards of Reporting Trials (CONSORT) statement [24].

### Participants

#### Recruitment

The target population for the trial are German adults with an indication for psychotherapy. Recruitment cooperation with the outpatient clinic of the Center for Psychosocial Medicine at the University Medical Center Hamburg-Eppendorf was instated. In addition, participants will be recruited through referrals from other cooperating outpatient facilities, physicians, psychotherapists, mailing lists, internet platforms, and social media.

#### Inclusion and Exclusion Criteria

Participants need to (1) be older than 18 years of age, (2) have an indication for psychotherapy, (3) have an email account and a web-connected device with a camera and a microphone, and (4) provide informed consent for study participation and the use of an audio record. The indication for psychotherapy will be operationalized by at least one suspected diagnosis according to the Diagnostic and Statistical Manual of Mental Disorders-fifth edition (DSM-5) [25].

Exclusion criteria are (1) a current outpatient or inpatient psychotherapy, (2) utilization of probatory sessions for psychotherapy within the last 4 weeks, (3) insufficient language comprehension, (4) insufficient attention performance or cognitive capacity to participate in the interviews and OIC, and (5) acute suicidality. Exclusion criteria (3) and (4) will be evaluated by the study psychologist during the telephone screening and the clinical interview. Based on a criteria-led graduated scheme, acute suicidality will be evaluated by the study psychologist within the clinical interview. In case of acute crises, further predefined steps for action will be initiated.

#### Sample Size

**Figure 1:**
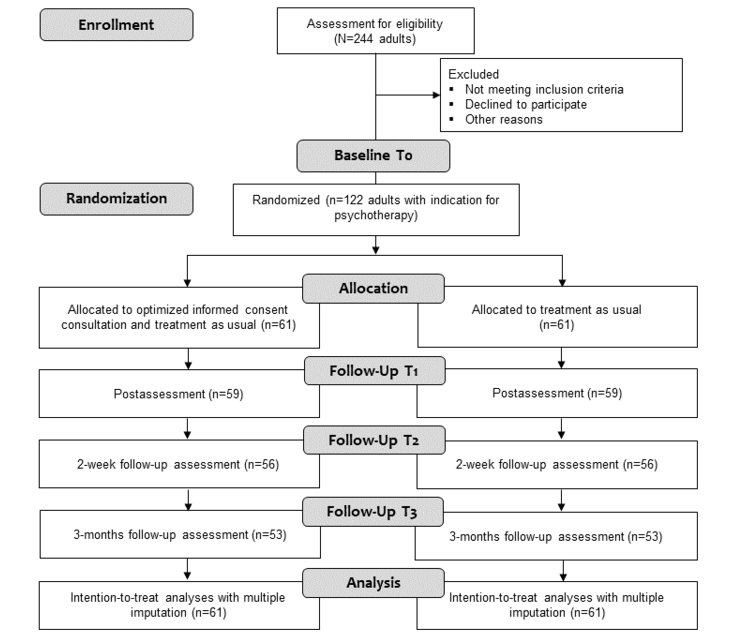
Anticipated study flow chart.

The required sample size was a priori calculated using the G*Power software. Based on a previous experimental study analyzing the effects of framing and personalizing information about endocrine treatment on side-effect expectations in healthy women [14], a small-to-medium effect size can be expected for the impact of OIC on the primary outcome (treatment expectations). For two-tailed testing and a predetermined α level of .05, 106 participants would provide 80% power to detect significant interaction and the main effects of Cohen *f*=0.125 on the primary outcome. To compensate for an anticipated dropout rate of 15%, a total sample of 122 participants will be randomly assigned to one of the 2 groups (n=61 per group). Since it is assumed that at least 50% of all individuals can be included in the clinical trial after screening, approximately 244 individuals will need to be screened (see Figure 1).

### Procedure

A telephone interview will take place before enrollment, in which oral study information will be given and interested persons are screened for self-reportable inclusion and exclusion criteria. If eligibility is given, interested persons will be invited for the first of the 2 web-based study visits that are conducted online by trained clinicians (Master of Science psychologists). At the first web-based study visit (T0), written information about the study will be given and the self-reportable inclusion and exclusion criteria will be queried. After providing their informed consent, participants will take part in a video-based Structured Clinical Interview for DSM-5 disorders (SCID-5) [26] to verify the indication for psychotherapy and check for exclusion criteria. If participants fulfill the eligibility criteria, the baseline assessment (T0) as well as the subsequent randomization will take place. At the end of T0, participants of both groups will receive TAU in form of an information brochure about psychotherapy. Participants will be invited to voluntarily study the brochure until the second web-based study visit (T1) 2 weeks later. At the second web-based study visit (T1), the intervention group will participate in the video-based OIC. Subsequently, all participants will take part in the postassessment and an interview for assessing the capacity to consent and adverse events. Upon request, participants will receive their individual results report of the SCID-5 within 1 week after T1. Two weeks (T2) and 3 months (T3) after T1, participants will be invited to complete 2 web-based follow-up questionnaires. A detailed summary of all the instruments is provided in Table 1.

**Table 1:** Schedule of enrollment, intervention, and assessment according to Standard Protocol Items: Recommendations for Interventional Trials (SPIRIT).

						Study period								
				Screening		Enrollment		Intervention		Postallocation				
				
Time point				–1		T0				T1		T2		T3
**Screening and enrollment**														
	Study information			✓		✓								
	Eligibility screen			✓		✓								
	Informed consent					✓								
	Randomization					✓								
**Intervention**														
	Treatment as usual + optimized informed consent consultation							✓						
	Treatment as usual							✓						
**Assessments**														
	**Primary end point**													
		Treatment expectations (TEX-Q^a^)			✓				✓		✓		✓	
	**Secondary end points**													
		Capacity to consent (MacCat-T^b^ interview)							✓					
		Decisional conflict (DCS^c^)			✓				✓		✓			
		Side effects of psychotherapy: occurrence expectations, anxiety, and expected coping (3 constructed items)			✓				✓		✓			
		Autonomous treatment motivation (ACMTQ^d^ subscale)			✓				✓		✓			
		Adherence intention (3 constructed items)			✓				✓		✓			
		Interest in and knowledge about psychotherapy in general (2 constructed items)			✓				✓		✓			
		Knowledge about what is meant by psychotherapy, its effectiveness, key mechanisms, side effects, legal and organizational aspects (5 constructed items)			✓				✓		✓			
		Information-seeking behavior toward finding a treatment (3 constructed items)									✓		✓	
		Utilization of treatment services (6 constructed items)									✓		✓	
		Satisfaction with received information (CSQ-8^e^)							✓					
		(Expected) adverse events (interview)							✓		✓^f^		✓^f^	
		(Expected) serious adverse events (interview)							✓		✓^f^		✓^f^	
**Modulators**														
	Psychopathology (suspected diagnosis; SCID-5^g^ interview)					✓								
	State anxiety (STADI^h^ subscale)									✓				
	Prior knowledge about psychotherapy (FPTM^i^ subscale)					✓								
	Prior psychotherapeutic experience (G-EEE^j^ subscale)					✓								
	Satisfaction with therapeutic relationship (HAQ^k^ subscale)					✓								
	Time spent with the information brochure (single item)					✓				✓				
	Sociodemographic characteristics (single item)					✓								
	Intake of mental health medication (SCID-5 interview)					✓								

^a^TEX-Q: Treatment Expectation Questionnaire.

^b^MacCAT-T interview: MacArthur Competence Assessment Tool for Treatment.

^c^DCS: Decisional Conflict Scale.

^d^ACMTQ: Autonomous Motivation for Therapy Scale.

^e^CSQ-8: 8-item Client Satisfaction Questionnaire.

^f^At follow-up (T2 and T3), (serious) adverse events will be assessed by self-report instead of an interview.

^g^SCID-5: Structured Clinical Interview for Diagnostic and Statistical Manual of Mental Disorders-fifth edition.

^h^STADI: State Trait Anxiety Inventory.

^i^FPTM: Questionnaire on Psychotherapy Motivation.

^j^G-EEE: Generic Rating for Treatment Pre-Experiences, Treatment Expectations, and Treatment Effects.

^k^HAQ: Helping Alliance Questionnaire.

### Study Intervention

OIC will be conducted online by trained study psychologists (Master of Science) at T1. For the purpose of conformity with a realistic preliminary psychotherapeutic consultation, OIC will last no longer than 35 minutes. OIC includes theory-overarching information about psychotherapy, clarifying what psychotherapy is, which forms and settings of psychotherapy exist, and how to get access to psychotherapy. The clinician will provide information about psychotherapeutic techniques, possible therapeutic objectives, the efficacy, and underlying mechanisms of psychotherapy. All 4 psychotherapeutic approaches that are recognized by the German social law (cognitive-behavioral psychotherapy, brief psychodynamic psychotherapy, psychoanalytic therapy, and systemic psychotherapy) are considered in OIC concerning their key mechanisms and techniques. In line with recent recommendations [23,27], OIC will contain information about the possible negative effects of psychotherapy (eg, the temporary increase of psychological strain) and respective individual coping strategies. Prior experience with psychotherapy and treatment expectations will be addressed since both are suggested to induce placebo and nocebo effects, which, in turn, might influence psychotherapy outcomes [7]. The clinician will target participants’ expectations about outcome (ie, treatment benefit, positive and negative effects), process (ie, expected satisfaction, side effects, own impact, behavioral control), and their coping with potential side effects. In accordance with ethical demands [7,23], OIC will provide a realistic and nondeceptive yet positive description of psychotherapy.

During OIC, the strategies of framing, contextualization, and shared decision-making will be applied. Framing strategies as described by Barnes et al [20] will be applied by embedding information about the possible negative effects in information about the overall effectiveness of psychotherapy [7,28]. Information will be formulated gain-framed. Contextualization will be used to adapt evidence-based information about psychotherapy to the individual information needs as well as the psychological and living conditions of the participant [6]. In line with the shared decision-making approach, participants will be informed about treatment alternatives (eg, psychopharmacotherapy) and will be invited to express their views and preferences [29]. Participants will be actively involved in the discussion of options. A balanced relationship between the clinician and the participant will be supported by an empathic attitude of the clinician. As a multimodal presentation might increase the comprehension of information and is assumed to elicit larger framing effects on the reduction of nocebo side effects [20], information will be given orally with additional support of visual information cards.

### TAU as a Comparator

To investigate the clinical significance of the newly developed OIC, a TAU condition will be used as a comparator. In both trial conditions, participants will receive an information brochure about psychotherapy from the Federal Chamber of Psychotherapists in Germany as TAU [30]. Participants may decide on their own whether and if so, how long they want to engage with the 80-page information brochure for psychotherapy patients.

### Randomization

Stratified permuted block randomization with a block size of 4 permutations will be used to randomize participants 1:1 to the OIC and TAU conditions. Stratification will be based on prior experience with psychotherapy (no vs positive vs negative prior experience) to ensure that individuals with heterogeneous treatment experiences are balanced in both arms. Before the first enrollment, the randomization sequences will be generated by a researcher who is not involved in the study conduction by using a web-based program. At the end of the first web-based study visit, the responsible study psychologist initially determines the type of prior experience with psychotherapy by evaluating the information given by each participant. The randomizing officer will then conduct the allocation of each pseudonymized study case according to the randomization plan. Finally, the randomizing officer will inform the respective study psychologist about the group membership on a case-by-case basis.

### Primary Outcome Measure

Participants’ treatment expectation will be assessed using the Treatment Expectation Questionnaire [31]. The Treatment Expectation Questionnaire is a generic self-rating scale assessing patients’ outcome and process expectations of medical and psychological treatments on 6 dimensions: treatment benefit, positive impact, adverse events, negative impact, process, and the behavioral control. The questionnaire consists of 15 items that are presented on an 11-point numeric rating scale. For all required analyses, mean subscale scores and the mean total score, each ranging from 0 to 10, will be used. Except for the subscales “adverse events” and “negative impact” with higher scores indicating lower treatment expectations, higher subscale scores indicate higher treatment expectations. Treatment expectation as the primary outcome will be operationalized by the total mean score because it combines process and outcome expectations. To investigate the potential effects of OIC, the impact of OIC on treatment expectations will be additionally analyzed for each of the 6 subscales following an exploratory approach. To counteract the problem of multiple comparisons requiring multiple simultaneous statistical tests, statistical inference will be adjusted using the Bonferroni-Holm correction to reduce the risk of α error inflation.

### Secondary Outcome Measures

The capacity to consent to treatment will be assessed by an adapted German version of the semistructured interview MacArthur Competence Assessment Tool for Treatment (MacCAT-T) [32,33]. Subscale scores, ranging from 0 to 6 (understanding), 0 to 8 (reasoning), 0 to 4 (appreciation), and 0 to 2 (choice), as well as the total sum score, ranging from 0 to 20, will be used for all required analyses. Higher scores indicate higher capacity to consent. Since the MacCAT-T provides a wide range of applications (eg, for dementia), the exact wordings of the questions have been rephrased and adapted to the psychotherapeutic context. Given that risks of psychotherapy may be diverse and multifaceted, it has been deemed sufficient if participants can name 1 risk of psychotherapy instead of 2, as demanded in the original form of the MacCAT-T. Thus, the maximum score of 2 in the item “understanding of benefits and risks” (subscale understanding) can be achieved, even if just 1 risk of psychotherapy can be expressed adequately. The ranges of all subscale scores as well as the range of the total sum score do not change due to this modification.

Decisional conflict will be assessed using the Decisional Conflict Scale [34,35]. The Decisional Conflict Scale consists of 16 items, which are divided into 5 distinct domains: uncertainty, informed, values clarity, support, and effective decision. All items are presented on a 5-point Likert scale, ranging from “not correct at all” to “fully correct.” A total score and 5 subscale scores, each ranging from 0 to 100, will be used for analyses, with higher scores indicating higher decisional conflict. The perceived support in decision-making for or against the utilization of psychotherapy is assessed by 1 self-developed item that is presented on an 11-point Likert scale, ranging from 0 to 10, with higher scores indicating greater perceived support.

Three additional items were developed in advance to assess participants’ (1) expectations about experiencing side effects of psychotherapy, (2) anxiety about experiencing side effects, and (3) expectations about coping with side effects. The corresponding items will be presented on an 11-point Likert scale, ranging from 0 to 10, with higher scores indicating higher expectation about occurring side effects, anxiety about side effects, or respective coping expectations.

The autonomous treatment motivation will be assessed by the translated subscale “autonomous motivation” of the Autonomous and Controlled Motivations for Treatment Questionnaire [36]. The translation from English to German was performed by a native English speaker. The subscale consists of 6 items, which are presented on a 7-point Likert scale, ranging from “strongly disagree” to “strongly agree.” The subscale mean score ranges from 1 to 7, with higher scores indicating higher autonomous treatment motivation, and will be used for all required analyses.

The adherence intention for psychotherapy will be assessed by 3 self-developed items, which will be presented on an 11-point Likert scale, ranging from “not sure at all” to “absolutely sure.” For all required analyses, the mean score will be used, ranging from 0 to 10, with higher scores indicating higher adherence intention.

Seven additional items were developed to assess (1) participants’ interest in psychotherapy as well as their (2) knowledge about psychotherapy in general, (3) what is meant by psychotherapy, (4) the effectiveness of psychotherapy, (5) key mechanisms of psychotherapy, (6) side effects of psychotherapy, and (7) legal and organizational aspects of psychotherapy. The corresponding items will be presented on an 11-point Likert scale, ranging from 0 to 10, with higher scores indicating greater interest or higher knowledge.

The satisfaction with received information will be assessed by an adapted version of the German version of the Client Satisfaction Questionnaire [37,38]. The word “treatment” will be replaced by “received information” to increase the specificity. The 8 items will be presented on a 4-point Likert scale and summed up to a total score, ranging from 8 to 32, with higher scores indicating higher satisfaction with received information.

The information-seeking behavior toward finding a treatment will be assessed by 3 self-developed items, which will be presented on a 7-point Likert scale, ranging from “strongly disagree” to “strongly agree.” The utilization of treatment services will be assessed by 6 self-developed items with the response options “yes” and “no.”

As recently recommended by Papaioannou et al [39], (expected) adverse events and (expected) serious adverse events of OIC and of study participation per se will be assessed at postassessment by using a short interview. In addition to open questions about individual adverse events, 3 a priori developed items about potential adverse events (feeling confused, feeling frightened about potential negative effects of psychotherapy, experiencing doubts about the decision to start psychotherapy) and serious adverse events (suicidal ideation, self-harm, hospitalization) will be assessed. Each event will be rated by the interviewer according to severity (5-point Likert scale) and its potential causal relationship to the study participation (5-point Likert scale). For follow-up assessments (T2 and T3), a self-report will be used instead of an interview.

### Blinding

Due to the nature of the study, neither participants nor the responsible study psychologists can be fully blinded toward the group membership. The assessment and analysis of the capacity to consent and the adverse events, however, will be carried out by researchers who are blinded regarding the randomization and do not otherwise interact with the respective participants throughout the entire course of the study. Questionnaire-based outcomes will be assessed pseudonymously via a web-based software, and the researcher conducting the statistical analyses will be blinded toward the randomized group allocation.

### Bias Control

To reduce heterogeneity in content and risk of performance bias, OIC was manualized and all responsible study psychologists have been specifically trained by a licensed psychotherapist for conducting OIC. A licensed psychotherapist will supervise the study psychologists regarding the conducting of OIC throughout the study. To increase the interrater reliability and to reduce the risk of experimenter bias, all interviewers conducting the MacCAT-T interview have been trained and will be blinded to group allocation. Based on an audio record, an independent and blinded interviewer will conduct a second rating of the MacCAT-T.

### Statistical Analyses

Linear mixed modeling for repeated measures will be conducted for the hypothesis referring to the primary outcome. Following the intention-to-treat approach, all randomized participants will be included in the analysis to avoid attrition bias. The model will contain one between-subject factor treatment (OIC + TAU vs TAU) and one within-subject factor time (T0, T1, and T2). Before linear mixed modeling, additional variables (eg, respective baseline scores, type of prior experiences with psychotherapy, prior knowledge about psychotherapy, satisfaction with the therapeutic relationship, state anxiety, the time of occupation with the information brochure) will be checked for significant associations with the respective outcomes to identify potential covariates. If there are additional covariates, they will be included in the linear mixed model. Group differences will be checked using Tukey posthoc tests. In case of imbalanced group sizes, Bonferroni-corrected posthoc tests will be carried out instead.

The analytical strategy of fitting linear mixed models will also be applied to all analyses, including secondary outcomes. Three exceptions for the described analytical strategy will be analyses including the secondary outcomes of capacity to consent, satisfaction with received information, and (serious) adverse events, for which group differences at postassessment will be examined by an independent sample two-sided *t* test, a Welch *t* test, or a Mann-Whitney *U* test. Intergroup differences at baseline will be detected using independent sample two-sided *t* tests, Welch *t* tests, or Mann-Whitney *U* tests (for continuous variables) and Pearson chi-square tests (for categorical variables). Partial eta squared will be reported for estimating the proportion of explained variance, with *η^2^*=0.01 indicating small, *η^2^*=0.06 indicating moderate, and *η^2^*=0.14 indicating large effects [40]. Cohen *d* will be determined as a measure of effect size for pairwise comparisons (standardized mean differences) by dividing the mean score difference between the groups to be compared by the pooled standard deviation. According to Cohen [40], values of *d*=0.2, *d*=0.5, and *d*=0.8 are considered to indicate small, medium, and large effect sizes, respectively.

Missing values will be imputed using the multiple imputation technique but only if more than 2% of the data is missing. In case of multiple imputation, analyses will be repeated based on per-protocol analyses as a sensitivity analysis. If less than 2% of the data is missing, no multiple imputation will be carried out and a sensitivity analysis will be conducted using the last observation carried forward method. Each hypothesis will be tested two-sided with an α level of .05. All statistical analyses will be performed using SPSS version 27 (IBM Corp).

### Ethics Approval

This trial is performed in accordance with the Declaration of Helsinki and was approved by the local ethics committee of the Center for Psychosocial Medicine, University-Medical Center Hamburg-Eppendorf, Hamburg, Germany (reference: LPEK-0292, 01.04.2021). The data generated from this study will be available for scientific purposes in PsychArchives (a disciplinary repository for psychological science) [41]. All participants provide informed written web-based consent for participation and for the publication of anonymized data for scientific purposes in the disciplinary repository.

## Results

The first participant was enrolled on August 5, 2021. We expect to complete data collection in December 2022. After data analysis within the first quarter of 2023, the results will be submitted for publication in peer-reviewed journals in summer 2023. Research reports will be additionally disseminated through scientific forums, including presentations at conferences.

## Discussion

The aim of this two-armed randomized controlled superiority trial is to evaluate the efficacy of a newly developed OIC for psychotherapy under consideration of its legal, ethical, and clinical functionalities. In this trial, the effects of an OIC combined with TAU is compared to those of TAU alone in a sample of German adults with an indication for psychotherapy. OIC was developed under consideration of the latest empirical evidence concerning the legal, ethical, and clinical requirements of a truthful informed consent procedure, including possible strategies to promote and integrate these functions. A semistructured guideline with supportive visual information cards was developed. Since OIC will take relatively little time and does not require advanced professional trainings, it might be a cost-effective tool for daily practice.

Noteworthily, a genuine clinical approach for integrating risk information into the informed consent for psychotherapy has been missing to this day. If proven effective, future patients might be adequately informed about possible risks of psychotherapy without violating the ethical principle of nonmaleficence. This multifaceted evaluation of a structured OIC might help reduce the initial reservations of psychotherapists about informing patients about the possible negative effects. Thus, OIC may represent the first feasible approach of fulfilling the legal demands of informed consent in clinical practice.

The web-based context was chosen to enable the participation of citizens living in rural areas with limited capacity of health care services. Since OIC is not restricted to a certain therapeutic approach, its scope of application is broad. In line with the current implications from psychotherapy research [42,43], OIC might contribute to a theory-overarching dissemination of recent empirical evidence into the care system.

Nevertheless, this trial will have some limitations that need to be acknowledged. Although the web-based context should help expanding the spectrum of recruitment, it might also lead to the exclusion of potential participants who do not have a suitable internet connection. Moreover, no conclusions can be drawn regarding the specific efficacy of each of the 3 applied strategies, namely, contextualization, framing, and shared decision-making. However, if identified as efficient, the impact of each subcomponent of OIC may further be analyzed on a factorial basis.

To this day, efforts to implement risk information into informed consent for psychotherapy seem to be rather insufficient. The newly developed OIC for psychotherapy might contribute to bridging the gap between theoretically assumed ideals of truthful informed consent and practical realities.

## Abbreviations

CONSORT: Consolidated Standards of Reporting Trials
DSM-5: Diagnostic and Statistical Manual of Mental Disorders-fifth edition
MacCAT-T: MacArthur Competence Assessment Tool for Treatment
OIC: optimized informed consent consultation
SCID-5: Structured Clinical Interview for Diagnostic and Statistical Manual of Mental Disorders-fifth edition
TAU: treatment as usual

## Data Availability

Data sharing is not applicable to this paper as no new data were created or analyzed in this study. The data sets generated during this study will be available in anonymized form for scientific purposes in the publicly available PsychArchives (a disciplinary repository for psychological science) [41].
